# Cholecystokinin enhances visceral pain-related affective memory via vagal afferent pathway in rats

**DOI:** 10.1186/1756-6606-5-19

**Published:** 2012-06-09

**Authors:** Bing Cao, Xu Zhang, Ni Yan, Shengliang Chen, Ying Li

**Affiliations:** 1 Neuroscience Laboratory, Department of Biology and Chemistry, City University of Hong Kong, Tat Chee Avenue, Kowloon, Hong Kong, China; 2 Department of Gastroenterology, Shanghai Institute of Digestive Diseases, Renji Hospital, Shanghai Jiaotong University, School of Medicine, 145 Middle Shandong Road, Shanghai, 200001, China

**Keywords:** Cholecystokinin, Conditioned place avoidance, Visceral pain, Visceral aversive memory, Anterior cingulate cortex, Vagal afferent pathway

## Abstract

**Background:**

Pain contains both sensory and affective dimensions. Using a rodent visceral pain assay that combines the colorectal distension (CRD) model with the conditioned place avoidance (CPA) paradigms, we measured a learned behavior that directly reflects the affective component of visceral pain, and showed that perigenual anterior cingulate cortex (pACC) activation is critical for memory processing involved in long-term visceral affective state and prediction of aversive stimuli by contextual cue. Progress has been made and suggested that activation of vagal afferents plays a role in the behavioral control nociception and memory storage processes.

In human patients, electrical vagus nerve stimulation enhanced retention of verbal learning performance. Cholecystokinin-octapeptide (CCK), which is a gastrointestinal hormone released during feeding, has been shown to enhance memory retention. Mice access to food immediately after training session enhanced memory retention. It has been well demonstrated that CCK acting on vagal afferent fibers mediates various physiological functions. We hypothesize that CCK activation of vagal afferent enhances visceral pain-related affective memory.

**Results:**

In the presented study, infusion of CCK-8 at physiological concentration combining with conditional training significantly increased the CRD-induced CPA scores, and enhanced the pain affective memory retention. In contrast, CCK had no effect on CPA induced by non-nociceptive aversive stimulus (U69,593). The physiological implications were further strengthened by the similar effects observed in the rats with duodenal infusion of 5% peptone, which has been shown to induce increases in plasma CCK levels. CCK-8 receptor antagonist CR-1409 or perivagal application of capsaicin abolished the effect of CCK on aversive visceral pain memory, which was consistent with the notion that vagal afferent modulates affective aspects of visceral pain. CCK does not change the nociceptive response (visceral pain sensitivity) and anterior cingulate cortex neuronal responses to CRD.

**Conclusion:**

CCK activating vagal afferent C fibers enhances memory consolidation and retention involved in long-term visceral negative affective state. Thus, in a number of gastrointestinal disorders, such as irritable bowel syndrome, nutrient content may contribute to painful visceral perception by enhancing visceral aversive memory via acts on vagal afferent pathway.

## Background

The conceptualization of pain in humans recognizes several interrelated components. The components involves the encoding and perception of stimulus parameters (e.g., stimulus localization, intensity, and quality), and the component involves the encoding of the affective salience or unpleasantness of the noxious stimulus [1]. Teasing apart the mechanisms that control the neural pathways mediating pain affect and sensation in nociceptive behavioral response is a challenge. Our series of publications have demonstrated that perigenual ACC (pACC) plays a critical role in the modulation of sensory aspect of visceral pain in viscerally hypersensitive rats [2-4]. The ACC has been proposed to process information relating to pain- unpleasantness, and contribute to the avoidance learning that sometimes follows as a secondary reaction to pain [5]. One of the first studies using a rodent visceral pain assay that combines the colorectal distension (CRD)-induced visceromotor response (VMR) with the conditioned place avoidance (CPA), we measured a learned behavior that directly reflects the affective component of visceral pain [6]. When CRD was paired with a distinct environment context, the rats spent significantly less time in this compartment on the post-conditioning test days as compared with the pre-conditioning day. Effects were lasted for several days. Lesions of pACC or administration of glutamate receptors antagonists abolished CRD-induced CPA; these data indicated that pACC activation is critical for the memory processing involved in long-term negative affective state and prediction of aversive stimuli by contextual cue [6].

Physiological and psychological events can either strengthen or weaken the formation of memories. Progress has been made and suggested that activation of vagal afferents plays a role in the behavioral control of nociception [7] and memory storage processes [8,9]. Clark et al [8] have shown that electrical vagus nerve stimulation (VNS) given immediately after training enhanced retention performance on an inhibitory-avoidance task in rats. In human patients VNS at an intensity similar to that effect in rodent enhanced retention of verbal learning (word recognition) performance [10]. Subdiaphragmic vagotomy attenuates the memory modulation produced by post-training systemic injections of various substances [11], including 4-OH amphetamine, leu-enkephalin [12], and substance P [13]. Cholecystokinin (CCK) is a gastrointestinal hormone released during feeding [14-16]. Electrophysiological and functional studies have demonstrated that CCK receptors in the vagal afferent fibers mediate various physiological functions [17-20]. Based on findings in rats and human, we hypothesize that CCK activation of vagal afferent facilitates the visceral pain memory consolidation involved in long-term negative affective state.

The current study used colorectal distension (CRD)-induced visceral pain with the conditioned place avoidance (CPA). We examined the learned behavior and aversive memory in conscious rats. In combination with CRD-CPA with administration of CCK this approach enables one to determine whether CCK modulates “aversiveness memory” of visceral pain. One question addressed in the current study concerns whether CCK-induced enhancement of affective component of pain memory is specifically with aversiveness of visceral nociceptor-activating stimuli, or is associated with aversive stimuli in general. We examined the effects of CCK on CPA induced by an aversive, but non-nociceptive-activating stimulus κ-opioid receptor agonist U69,593 [21]. To identify the role of vagal afferent *C*-fibers in the mediation of CCK’s action perivagal capsaicin studies were performed. To determine if endogenously released CCK also acts on capsaicin-sensitive vagal afferent to enhance pain memory, we showed that the similar effects were observed following intra-duodenal perfusion of peptone, which has been shown to postprandial release endogenous CCK [16,22]. The nociceptive response (visceromotor response [VMR]) to CRD was recorded to clarify that administration of CCK does not alter the visceral pain sensitivity. Our recent studies showed that ACC activation was critical for the memory processing involved in visceral long-term negative affective state [6], however, electrophysiological recording of pACC neurons showed that administration of CCK did not change the ACC responses to CRD.

## Results

### Effects of CCK-8 on colorectal distention induced conditioned place avoidance (CRD-CPA)

Pain contains both sensory and affective dimensions. Except for human experiments where self report is possible, teasing apart the mechanisms that control the neural pathways mediating pain affect and sensation in an overall animal nociceptive behavioral response is a challenge. We measured a learned behavior that directly reflects the affective component of visceral pain (CRD-induced CPA) [6]. We hypothesize that CCK activation of vagal afferent enhances visceral pain-related affective memory.

**Figure 1:**
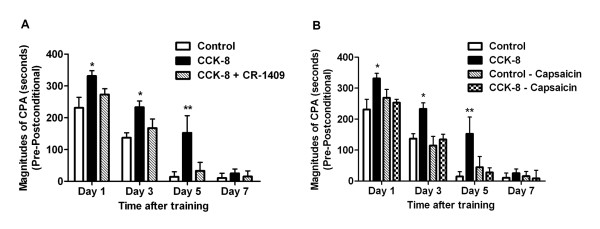
**CPA in control and CCK-8 infusion rats with or without CCK-A-R antagonist or capsaicin pretreatment. (A)** For the CPA score the amount of time spent in the conditioning compartment on the post-conditioning days were subtracted from the amount of time spent in the same compartment on the pre-conditioning day. The rats spent less time in this compartment on the post-conditioning test day as compared with the pre-conditioning test day. Compared with control rats, intravenous infusion of CCK-8 after conditioning training markedly increased the CPA scores in post-conditioning day 1, 3 and 5, respectively. This effect was blocked by pretreatment of CCK-A receptor antagonist CR-1409. **(B)** Perivagal capsaicin treatment in control rats with saline infusion had no effect on CPA scores but eliminated the enhanced CRD-induced CPA in rats with CCK-8 infusion. For easy and clear comparison, the same control group and CCK-8 group were used here as in Figure 1 A. Results were presented as means ± SE. Statistical significance was determined by Two-way Repeated measurement ANOVA followed by Bonferroni posttests. * P < 0.05, ** P < 0.01 compared between CCK-8 treatment group and control group, n=5 for each group.

In all groups of rats, no initial preference for any of the 3 compartments in the place conditioning apparatus was detected on the pre-conditioning test day. When CRD (40 mm Hg) was paired with a particular compartment in the place-conditioning apparatus all rats spent significantly less time in this compartment on the post-conditioning test days compared with the pre-conditioning test day. In control rats (with saline infusion), significant effects on the magnitudes of CPA scores (pre-conditioning minus post-conditioning) were lasted for 3 days. However, the rats with intravenous infusion of CCK-8 (40 pmol/kg/h for 15 min) immediately following CRD-conditioning training displayed significant higher magnitudes of CPA scores. The significant effects lasted for 5 days compared to 3 days in the control rats. The scores of rats in these studies are shown in Figure 1A. (F_time (3,36)_ =63.09, F_treatment (2,36)_ =13.45; n=5 for each group, Figure 1A). These observations suggest that post-training administration of CCK enhances the aversive emotional responses of visceral pain, and further prolongs the visceral pain memory. Administration of CCK-A receptor antagonist CR-1409 (10 mg/kg iv) abolished the effects of CCK on pain memory. The magnitudes of CPA scores were reduced (Figure 1A).

To identify the sites of action of CCK to enhance visceral pain memory we performed perivagal capsaicin study, which abolished vagal nerve *C* fibers [17,20,23,24]. Perivagal capsaicin treatment had no effect on the magnitudes of CPA scores in the control rats. In contrast, perivagal application of capsaicin eliminated the effects of CCK on facilitating CRD-induced aversive aspect of visceral pain memory (Figure 1B) suggesting CCK acts on vagal capsaicin-sensitive C-fibers to enhance visceral aversive pain memory.

### Effects of peptone perfusion on visceral pain-induced conditioned place avoidance (CPA)

**Figure 2:**
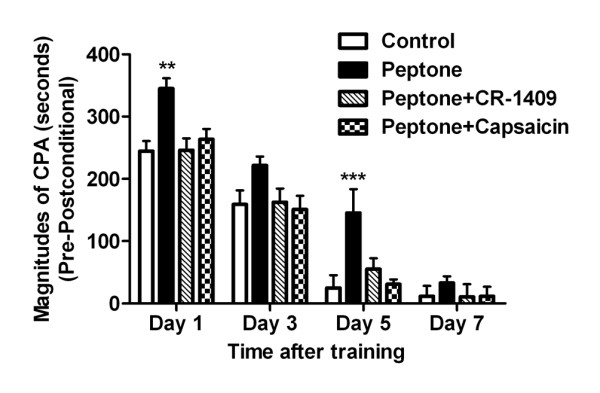
**CPA in control and peptone infusion rats with or without CCK-A-R antagonist or capsaicin pretreatment.** For the CPA score the amount of time spent in the conditioning compartment on the post-conditioning days were subtracted from the amount of time spent in the same compartment on the pre-conditioning day. Compared with control rats, intraduodenal infusion of 5% peptone (3 ml in 15 min) after conditioning trainings significantly increased CPA scores in post-conditioning day 1 and 5, respectively. The enhanced effects of peptone infusion on CRD-induced CPA were blocked by pretreatment of CCK-A receptor antagonist CR-1409 or perivagal capsaicin application. Results were presented as means ± SE. Statistical significance was determined by Two-way Repeated measurement ANOVA followed by Bonferroni posttests. ** P < 0.01, *** P < 0.001 compared between peptone infusion group and control group, n=5 for each group.

Although exogenously infused CCK facilitates CRD-induced aversive aspect of visceral pain memory, the physiological implication of postprandial released CCK is unclear. Previously, we have shown that intraduodenal perfusion of 5% peptone increased plasma CCK levels by stimulating CCK-releasing peptide (CCK-RP) secretion [16]. Here, we reported that intra-duodenal infusion of 5% peptone (3ml for 15 min) after CRD training increased the magnitudes of CPA scores (Figure 2). The significant effect maintained for 5 days (F_Time__(3, 48)_ =165.38, F_Treatment__(3, 48)_ = 9.29; n=5 for each group). Administration of CCK-A receptor antagonist CR-1409 (10 mg/kg i.v.) abolished the effects of intra-duodenal perfusion of peptone. The CR-1409 treated rats displayed significantly lower magnitudes of CPA scores (Figure 2). Further, perivagal capsaicin pretreatment eliminated the effects of peptone on the increases of the CPA scores induced by visceral pain simulation (Figure 2). These observations indicate that intra-luminal peptone release CCK, which acts on capsaicin-sensitive vagal afferent fibers to enhance visceral pain affection and prolong the aversive pain memory.

### Effects of CCK-8 on U69,593-induced CPA

To clarify whether the effects of CCK-8 on enhancing pain memory are specifically associated with the aversiveness of visceral nociceptor-activating stimuli or with aversive stimuli in general, we examined the effects of CCK on CPA induced by an aversive, but non-nociceptive-activating stimulus.

**Figure 3:**
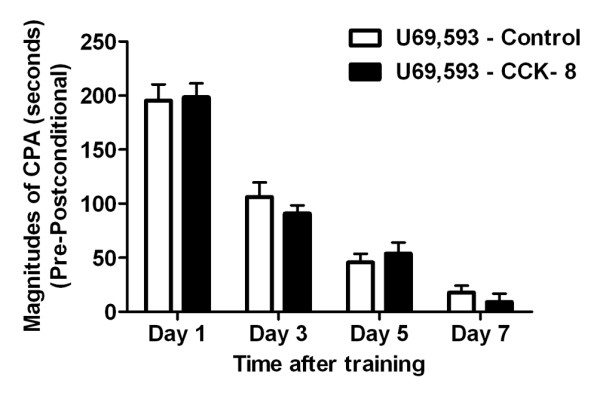
**CPA produced by the κ-opioid receptor agonist U69,593 in control and CCK-8 infusion rats.** For the CPA score the amount of time spent in the conditioning compartment on the post-conditioning days were subtracted from the amount of time spent in the same compartment on the pre-conditioning day. Compared with control rats, no significant differences of CPA scores were observed in the rats with post training CCK-8 infusion. Results were presented as means ± SE. Statistical significance was determined by Two-way Repeated measurement ANOVA followed by Bonferroni posttests, n=5 for each group.

Mu-opioid receptor agonists function as rewarding stimuli, whereas agonists at kappa-opioid receptors induce aversive states. These motivational effects have been attributed to interactions of exogenous opioids with endogenous reward pathways in the brain. The kappa-opioid receptor agonist U69,593, which is known to be aversive [21] when injected systemically, was administrated (s.c.) and paired with a distinct compartment in the apparatus. The conditioning procedure was similar to that used in the CRD-induced CPA. The scores of rats in these studies are shown in Figure 3. Unlike the CRD-induced CPA (Figure 1A), post training CCK-8 administration did not change the systemic U69,593-induced CPA responses profiles (Figure 3). Therefore, post training CCK-8 administration did not have a general enhancement on learning in the place-conditioning paradigm, but it markedly increases the magnitudes of aversiveness of visceral pain, and prolongs the visceral pain memory.

### CCK-8 and peptone administration did not affect visceromotor responses (VMR) to distension

To clarify if post-training administration of CCK-8 or peptone increase the sensory aspect of visceral pain (pain sensitivity), which results in facilitation of affective aspect of pain memory, we tested the CRD-induced visceromotor response after CCK-8 or peptone administration. The VMR induced by CRD is a brain stem-mediated reflex contraction of the abdominal musculature.

**Figure 4:**
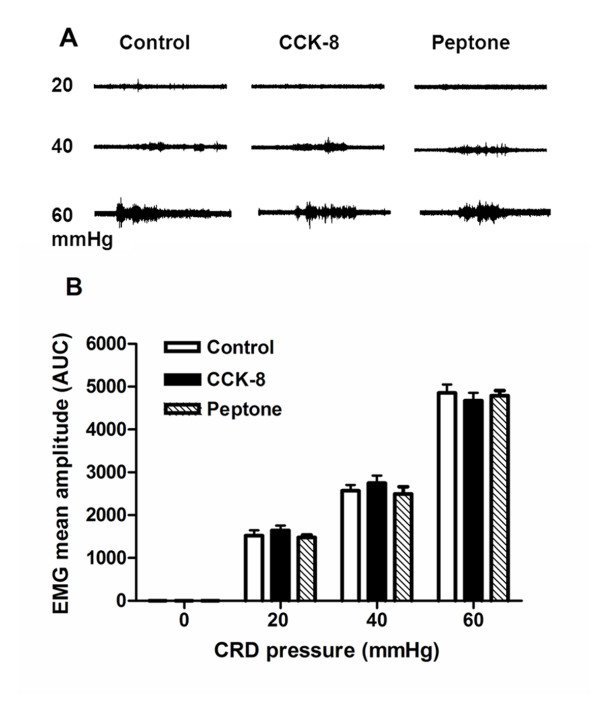
**VMR to graded pressure CRDs in control rats, CCK-8 infusion and 5% peptone infusion rats. (A)** Representative abdominal muscle electromyograms of the VMR to graded-pressure CRD (20, 40 and 60 mmHg) recorded from the external oblique pelvic muscle in normal rats and rats with CCK-8 and peptone infusion. No obvious difference was found among the three groups. **(B)** Histograph showed the effects of CCK infusion and 5% peptone infusion on the VMR to graded-pressure CRD in rats. Mean amplitude of the abdominal muscle contraction expressed as AUC after baseline subtraction was presented. No significant difference was found among the three groups. Results were expressed as means ± SE. Statistical comparisons of the VMR in various groups were made by two-way repeated-measures ANOVA, followed by multiple comparisons adjusted by the Bonferroni test, n=5 for each groups.

In response to stimuli that cause pain, all animals show musculoskeletal and autonomic responses, the so-called pseudoaffective reflex responses [25]. The results are shown in the Figure 4. Compared to the control group administration of CCK-8 or intra-duodenal perfusion of peptone had no effects on VMR to colorectal distension. These observations indicated that the roles of CCK to enhance visceral pain memory were not mediated by altering the behavioral visceral pain responses (pain intensity) in the rats under physiological condition.

### ACC neuronal responses to colorectal distension (CRD)

Our series of published observations characterized neural electrophysiological activity of ACC during processing of visceral nociceptive stimulation [3,4,26]. It has been well documented that the ACC is involved in pain processing and encoding of negative affects in humans, which results in pain-related unpleasantness. Recently, performing CRD-induced CPA studies we demonstrated that perigenual ACC activation is critical for the memory processing involved in long-term visceral negative affective state [6]. In this study to examine whether CCK alters the ACC neuronal responses induced by visceral nociceptive stimulation, which in turn to enhance aversive pain memory, we performed electrophysiological recording of ACC neurons in response to CRD in the vehicle infusion and in combined with CCK-8 infusion.

#### Control

**Figure 5:**
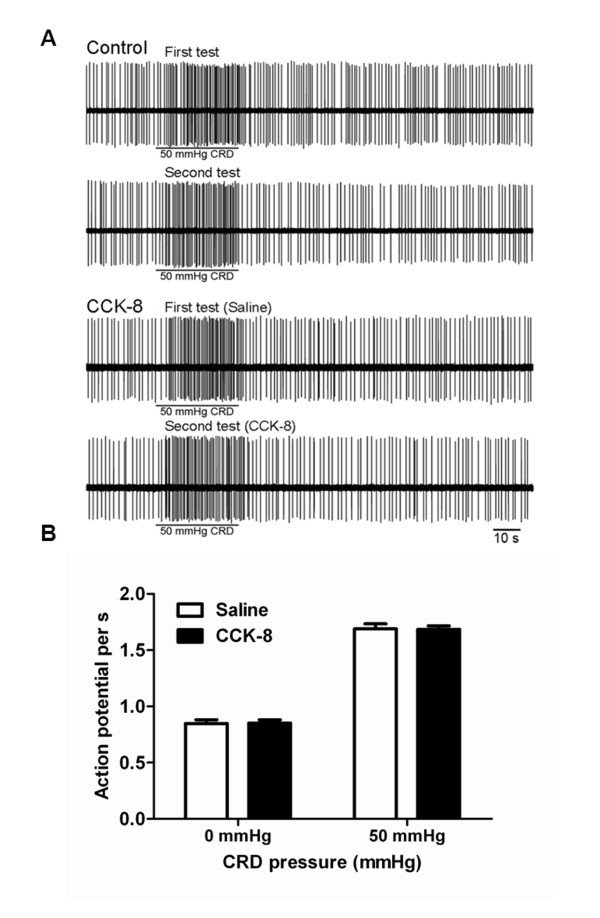
**Neuronal activities of CRD-excited ACC neurons in response to CRD in CCK-8 infusion rats. (A)** Recording of a 50 mmHg colorectal distension (CRD)-excited ACC neuron in a control rat and a rat with CCK-8 infusion. In control rats, the responses to 50 mmHg CRD were tested twice (first test and second test) and the results showed that the responses were stable and repeatable. In CCK-8-treated group, CCK-8 infusion did not change ACC spontaneous neuronal firing and the spike firing rates in response to 50 mmHg CRD compared with saline infusion.** (B)** Histograph shows that the spontaneous firing of CRD-excited neurons of ACC and spike firing rates in response to CRD pressures (50 mmHg) was not changed in rats treated with CCK-8 compared with saline. Results were expressed as means ± SE. Statistical comparisons between control and CCK-8-treated rats were made by Student’s t-test.

Total of 25 rats were examined for their response to CRD (50 mmHg) with intravenous (i.v,) saline infusion. One neuron per rat was labeled with neurobiotin. Neurobiotin labeling failed in 4 of 25 rats. Of these 4 rats, the juxtacellular configurations were lost during the injection in three rats, and histological studies revealed an absence of stained cells in 1 rat, presumably because neurobiotin was deposited extracellularly. These rats were excluded from further study. Histological localization of the CRD-responsive neurons showed that the recording electrodes were successfully placed in the ACC of 21 rats. All of the recordings had uniform spike amplitude and could be minimized and separated from the recordings of the neighboring neurons. From these 21 rats seventy four neurons were recorded. ACC spontaneous activity was monitored for 2 min to confirm the stability of basal firing frequency. Basal ACC neuronal firing rate was assessed over 30s to quantify the resting discharge. Among the 74 neurons, 50 showed no response to CRD (67.5%), and were referred to as CRD-non-responsive; twenty one of 74 neurons (28%) exhibited an excitatory response characterized by increased spike firings from baseline 0.862 ± 0.036 to 1.680 ± 0.041 and thus were referred to as CRD excited neurons. Histological localization of the CRD-excited neurons showed that they were located in the cingulate cortex area 1 (Cg1) and the prelimbic (PrL) cortex. Three of 74 neurons were inhibited by 50 mmHg CRD (CRD-inhibited neurons). These CRD-inhibited neurons were not tested further. Consistent with our previous observation [26], in the current study, the CRD-excitatory neurons responding to 50 mmHg CRD were tested twice and the results showed that the responses were consistent and repeatable (Figure 5A).

**Figure 6:**
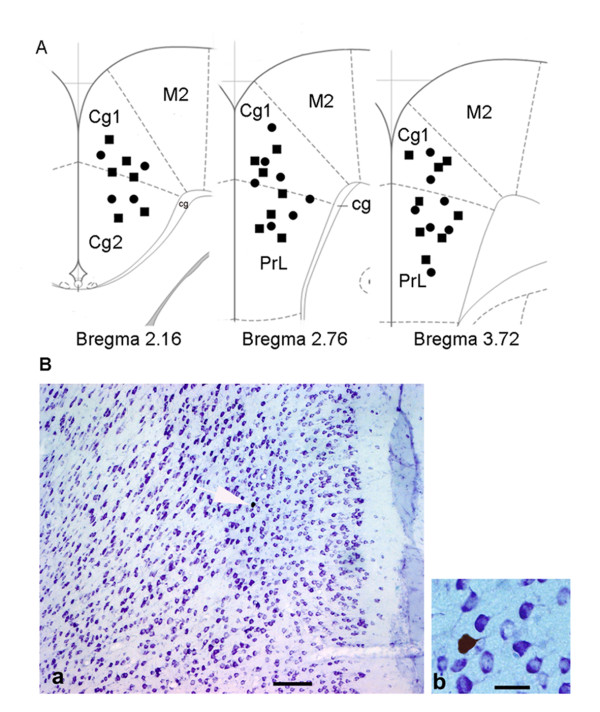
**Location and labeling of identified anterior cingulate cortex neurons with neurobiotin. (A)** Location of neurons recorded in the anterior cingulate cortex (ACC). Coronal sections from the caudal to rostral regions of the ACC (i.e., bregma 2.16, 2.76, 3.72 mm) show the electrophysiological recording sites. Squares and circles indicate CRD-excited ACC neurons in control rats and CCK-8 treated rats, respectively. Cg, cingulum; Cg1, cingulate cortex, area 1; Cg2, cingulate cortex, area 2; M2, Secondary motor cortex; PrL, prelimbic cortex. (B) Microphotograph of neurons in the ACC labeled with neurobiotin a, thionine-stained coronal section shows the distribution of CRD-excited neurons in the ACC. b, higher magnification of the neurobiotin-labeled pyramidal ACC neuron in a (arrow). Scale bars: a, 250 μm; b, 50μm.

#### ACC neuronal responses to CRD in rats with CCK-8 infusion

A total of 28 rats with CCK-8 infusion were studied for the spontaneous firing and the response to 50 mmHg CRD in ACC. On completion of the experiment, during the injection of neurobiotin, the juxtacellular configurations were lost in three rats. Histology studies revealed an absence of stained cells in one rat. The data from these animals were not presented. Recording electrodes were successfully placed in the ACC of 24 rats in the cingulate cortex area 1 (Cg1) and the prelimbic cortex (Figure 6A). From these 24 rats, forty three neurons were identified. Three types of neurons were classified according to their CRD response: 19 of 43 (44%) were CRD-non-responsive and 24 of 43 (56%) were activated by CRD. Six of 24 neurons were inhibited by 50 mmHg CRD (CRD-inhibited neurons). These CRD-inhibited neurons were not tested further. Of the 24 CRD-activated neurons, 18 of 24 neurons were 50 mmHg CRD-excited neurons. The ACC spontaneous neuronal firing and spike firings in response to 50 mmHg CRD with saline infusion were 0.847 ± 0.034 and 1.690 ± 0.045 spikes/s, respectively. When simultaneously infused with CCK-8, the average spontaneous activity recorded in the CRD-excitatory neurons was 0.840± 0.03 spikes/s. The ACC spike firings in response to 50 mmHg CRD increased to 1.662 ± 0.045 spikes/s. Original action potential recordings are presented in Figure 5A. Mean neuronal firing frequencies are shown in Figure 5B. These results suggest that compared with saline infusion CCK had no effects on ACC neuronal spontaneous activity, and did not alter the neuronal firings in response to colorectal distension. Therefore, it appears unlikely that the enhancement of pain memory by CCK infused following conditional training is mediated by modulating ACC neurons activations.

## Discussion

Pain contains both sensory and affective dimensions. Studies of the perception of unpleasantness are only amenable to human experiments where self-report is possible, whereas the neural substrates of learning and motivated behavior are more easily studied in animals. Recently, we used behavioral paradigms visceromotor responses (VMR) evoked by colorectal distention (CRD) to assess visceral pain in the conscious rat [2,3]. Using such tasks and models, we have shown that CRD induced conditioned place avoidance (CPA) when paired with a distinct environment context [6]. In conditioned avoidance procedures, animal avoids stimuli based on the formation of a negative association between the given stimuli and the environment. This tendency for animal to avoid environmental cues that have been deemed aversive is believed to be affective important [27].

Current study used three days conditioning protocol; we were able to show that a negative affective state was maintained for 3 days following acute visceral pain insult in the absence of further conditioning. These results are consistent with the notion that acute pain insult leads to the persistence of negative affective state. It is particularly interesting in light of clinical data, which indicates how the emotional or motivational effects of visceral pain may live much longer than the pain itself [28].

Physiological and psychological events can either strengthen or weaken the formation of memories. In the animal and human, available evidences indicated that vagal nerve stimulation enhanced memory and attention [8,10,29]. Several studies have shown that subdiaphragmic vagotomy attenuates the memory modulation produced by post-training systemic injections of various substances, including 4-OH amphetamine, Leu-enkephalin [12], and substance P [13]. These findings suggest that peripherally acting substances may modulate memory storage processes via the activation of peripheral receptors that send neural messages to the brain via the vagus nerve.

An elegant study have demonstrated that when mice were fed immediately after aversive T-maze training, they remembered that task better than when access to food was delayed and intraperitoneally administration of CCK-8 enhanced memory retention in the mice after aversive training [19]. Similarly, when humans are fed immediately after learning, they have better recall of a complex task 48 hours later. Children who have a 63% increase in the amount eaten at breakfast have enhanced short-term memory [30]. Cholecystokinin-octapeptide (CCK-8), is a gastrointestinal hormone released during feeding [16,22]. Electrophysiological studies in rats have provided evidence that CCK stimulates vagal afferent [18,23] to induce satiety [31] and decrease gastric emptying [17].

The formation of memories was strengthened by physiological and psychological events. Some of these events may occur immediately before or during the perception of a stimulus, enabling an organism to learn about it more efficiently [32]. In contrast, arousal may also occur shortly following a learning experience, that is, during memory consolidation [33]. The current study demonstrated that intravenous infusion of CCK at physiological doses [20] immediately following a visceral aversive learning experience (colorectal distension), improved conditional place avoidance performance in rats suggesting that activations of CCK during the memory consolidation period were capable to enhance memory storage, rather than the acquisition, of the information. The increases in CPA scores maintained for 5 days compared with 3 days in the rats subjected to sham treatment. The higher CPA scores throughout the post-conditioning days suggests the higher degree of aversiveness, which may mimic the serious negative affective state in human and decrease pain tolerance.

The vagus nerve, like all cranial nerves, contains three types of fibers (A–C), distinguished by their physical and electrical conductance properties. We have demonstrated previously, that subdiaphragmatic electric vagal stimulation reduced pain [7], this effect only occurred through stimulation of Aδ-fibers and was absent when C-fibers were stimulated. Capsaicin is a specific activator of the transient receptor potential vanilloid type 1 (TRPV1) channel, a polymodal nociceptive transducer expressed predominantly in non-myelinated sensory afferents. Perivagal application of capsaicin has been shown to inhibit axonal transport of peptides including substance P and somatostatin [24,34]. Our published data have demonstrated that CCK-8 at physiological levels stimulated vagal afferent neurons, which in turn stimulated pancreatic secretion via a vago-vagal reflex [20,35]. Perivagal capsaicin treatment abolished CCK-8 stimulated vagal afferent neuronal responses and pancreatic secretion [20,23,24,35]. This treatment also suppressed the vagal neuronal responses induced by secretin and 5-HT [18,36]. To verify that perivagal application of capsaicin was effective in ablating capsaicin-sensitive vagal afferent fibers, using retrograde tracing, we showed that perivagal capsaicin treatment prevented uptake of True blue in the nodose ganglia [24]. In the current study perivagal application of capsaicin abolished the effects of CCK and peptone on the enhancement of affective pain memory retention suggesting CCK acts on capsaicin-sensitive vagal afferent *C*-fibers to enhance pain memory.

In current study, we addressed a question concerning whether CCK modulate CRD-CPA by altering the visceral pain sensation. Behavioral visceral pain responses were examined. We showed that the rats subjected to immediately post-conditioning CCK infusion or sham treatment exhibited pressure-dependent increases in the CRD-induced VMR. These responses were not significantly changed after CCK administration suggesting CCK had no effects on the CRD-induced pain responses. Therefore, it is highly unlikely that the enhanced retention of CPA performance observed following CCK was due to alteration of the visceral pain sensitivity.

The findings of this study raise the question as to what physiological stimuli activate the vagal afferents that produce this modulation of visceral pain affection. To clarify if endogenously released CCK produce similar effects we performed study of intra-duodenal perfusion of peptone. In the rat, protein is the major dietary intestinal stimulus for CCK release. We have characterized that the release of CCK into the circulation is mediated by a “CCK-releasing peptide” secreted into the intestine of the rat [22]. We showed in this study that intraduodenal infusion of peptone during conditioning training enhanced the pain memory retention. The effects were similar to the effects of exogenous CCK administration, and were abolished by CR-1409 suggesting postprandial released CCK played a role in modulating pain affective memory.

The anterior cingulated cortex (ACC) is a major cortical component of the limbic loop system, and its functional relationship to emotional and motivational responses have been well described [5]. Performing single ACC neuronal activities in response to colorectal distension (CRD) our previous works have identified CRD-responsive neurons in the ACC and showed that persistence of a heightened visceral afferent nociceptive input to the ACC induced ACC sensitization [26]. Moreover, recent studies demonstrated that neurons in the pACC are necessary for the “aversiveness” of visceral nociceptor stimulation [6]. Since ACC activation is critical for the memory processing involved in long-term visceral negative affective state, it is important to clarify whether ACC is involved in the CCK-induced enhancement of pain aversive memory. In the present study, to determine if CCK enable to modulate ACC neuronal activities we performed electrophysiological recording of single ACC neuron in in vivo. We confirmed our previous reports that rostral ACC neurons were activated by CRD pressure-dependently. However, administration of CCK did not change the basal ACC neuronal firings. Simultaneously infusion of CCK during colorectal distension (CRD) had no effects on CRD-induced ACC neuronal responses. Thus, CCK enhances the visceral pain affective memory retention; this process appears not to be mediated by alteration of ACC neuronal responses to visceral noxious stimuli.

The most robust activation detected by fMRI following vagus nerve stimulation was seen in the thalami and insular cortices suggesting that these areas may play a role in modulating cerebral cortical activity [37]. Currently, the mechanisms and neuronal circuitry responsible for mediating CCK-induced enhancement of visceral pain related aversiveness memory are unclear. The pain is likely to be reflected in a matrix of neuronal structures rather than in a fixed pain center. A ‘neuromatrix' incorporating, for example, the ACC and the prefrontal and insula cortices, amygdala and hippocampus may be involved in the processing of pain memory without any single region unto itself being necessary and sufficient for the pain experience [38].

Our studies support the theory of the ongoing nature visceral pain-induced affective disorder observed in the clinic, such as the irritable bowel syndrome (IBS). Altered vagal function in patients with IBS has been reported. Findings in the current study, that feeding facilities visceral pain-related affective memory, underscores the importance of memory in visceral pain perception [3,28].

## Conclusions

Vagal afferent modulates negative affective aspects of visceral pain. At physiological condition CCK activating vagal afferent C fibers enhances visceral pain-related affective processing and memory. Thus, in a number of gastrointestinal disorders, such as IBS, gastrointestinal nutrient content may contribute to painful visceral perception by enhancing visceral aversive memory via released hormones, which act on vagal afferent pathway.

## Methods

### Ethical approval

All experimental protocols procedures were approved by the City University Committee on Use and Care of Animals, and the Department of Health Hong Kong. All chemicals were purchased from Sigma-Aldrich (St Louis, MO). Experiments were performed on adult male Sprague–Dawley rats (250–300 g). For surgical preparations, rats were anesthetized with a mixture of xylazine and ketamine according to the protocol described in our previous publication.

### Conditioned place avoidance (CPA)

Place conditioning apparatus consisted of three wooden compartments. One conditioning compartment had horizontal stripes on the walls and an odor of 1.0% acetic acid, whereas the other had vertical stripes and standardized cinnamon scent associated with it. Walls of uniform color characterized and no distinctive odor characterized the neutral compartment. The experimental process consists of three distinct sessions: a pre-conditioning session (days 1), conditioning session (day 2–4), and post-conditioning session (e.g., test days, 1, 3, 5 and 7 days after conditioning day) as we described previously [6]. On the first day, rats were individually placed in the neutral compartment and were allowed to explore the two conditioning compartments.

#### Pre-conditioning day (day 1)

On day 1, the entrance connected to each compartment was opened. Rat was allowed to move freely throughout the entire apparatus (i.e., all three compartments) for 20 min. The times spent by the rat in each compartment were recorded. Animal spending more than an 80% (time spent >16 min) or less than 20% (time spent < 4 min) of the total time in a chamber were eliminated from further testing (approximately 15% of total animal).

#### Conditioning days (days 2–4)

The conditioning phase of all experiments consisted of 3 days. In the morning, rats received nothing, and were randomly confined with one of the compartment for 45 min. In the afternoon, rats received treatment being paired with CRD 40 mm Hg or CRD 0 mm Hg in the other conditioning compartment for 45 min. A polyethylene tube (i.d., 1.67 mm) attached to a balloon (length, 40 cm) lightly coated with a surgical lubricant was placed in the colon and secured to the base of the tail. Colorectal distension (40 mm Hg) was produced by rapidly injecting saline into the colonic balloon over 1 second and maintaining the distension for 30 seconds with 3-min interval, and repeated five times. The 0 mm Hg CRD was served as sham treatment.

In separate groups of rats the effects of s.c. injection of U69,593 (a ĸ-opiofid receptor agonist) or s.c. vehicle were paired with a distinct compartment in a place conditioning apparatus.

#### Post-conditioning days (1, 3, 5 and 7 days after conditioning day, test days)

The same trial was performed as pre-conditioning session (day 1). Each rat was allowed to move freely throughout the three compartments for 20 min with no aversive stimulus (CRD) presented. The time spent in each compartment was recorded.

### Intravenous infusion of CCK-8

After rats were anesthetized with xylazine and ketamine (13 and 87 mg/kg body wt, respectively), a 2-cm-long heck incision was made, a polyethylene catheter (PE-10; Clay-Adams PE 10,, Becton Dickinson, Sparks, MD) was inserted into the external jugular vein and routed through subcutaneously to the back of the neck for IV infusion using a syringe-driven pump. The rats were allowed to recover; the CCK studies were performed 5 days after surgery. In our previous works we have clarified that intravenous infusion of CCK-8 at dose of 40 pmol/kg/h produced plasma CCK concentration mimic the postprandial plasma CCK levels [20]. Therefore, in the present studies, in a group of rats, CCK-8 (40 pmol/kg/h) was infused for 15 minutes (a 250 g rat received a dose of around 2.5 pmol of CCK-8) immediately following conditioning training. The CPA studies were conducted as described above.

### Intra-duodenal perfusion of 5% peptone

After rats were anesthetized with xylazine and ketamine, through a midline incision, a polyethylene cannula (Clay-Adams PE-50) was placed into the duodenum slightly above the sphincter of Oddi, and routed through the abdominal wall and subcutaneously to the back of the neck for intra-duodenal perfusion. The rats were allowed to recover; studies were performed 5 days after surgery. Immediately following conditioning training, rats received an intra-duodenal perfusion of 3 ml phosphate-buffered saline or 3 ml 5% peptone (mol wt<1000, pH 6.0, osmolality 300 mOsm/liter) for 15 min using a syringe-driven pump. The 5% peptone test solution was prepared by ultrafiltration using an Amicon membrane (YM1) and the filtrate which only contained peptides with mol wt <1000 was used as a test solution. Luminal perfusion peptone has been shown to release CCK by stimulates CCK-releasing peptide secretion [16,35].

### Perivagal application of capsaicin

To investigate the role of vagal afferent pathway in the mediation of CCK's action, we examined the effects of perivagal application of capsaicin as we described previously [20,23,24]. Before surgery, atropine (0.5 mg/kg ip) was administered to reduce the acute effects of capsaicin on the cardiovascular and respiratory system. After rats were anesthetized with xylazine and ketamine, a 3-cm-long midline laparotomy incision was made through the abdominal wall. The esophagus and the abdominal vagal trunks were exposed and a piece of parafilm lamina was placed beneath them to minimize the spread of capsaicin to surrounding tissues. A cotton pledget soaked in capsaicin solution (1mg of capsaicin dissolved in 1ml of vehicle (10% Tween 80 in olive oil)) was placed around the esophagus for 30min. Capsaicin drops (0.1 ml per rat) were applied every 5 min to keep the cotton moist. The surgical procedure for the control animals was identical except that only the vehicle for capsaicin (10% Tween 80 in olive oil) was perivagally applied. The area was then thoroughly rinsed with saline and dried with sterile swabs, and stitching was performed to help to close the wounds. The CRD-CPA studies combined with administration of CCK were performed 5 days after perivagal application of capsaicin. Rats were checked for normal eye wiping movement, which indicates that of perivagal application of capsaicin has no systemic effect. To demonstrate that perivagal application of capsaicin was effective in ablating capsaicin sensitive vagal afferent fibers, in our previous studies [24], we performed retrograde tracing studies. An aqueous suspension of fluorescent dye, True blue was injected into the anterior wall of the stomach. Animals were killed 6 days after injection for localization of the fluorescent dye. The nodose ganglia were surgically removed; substance P immunohistochemistry studies were performed for the localization of substance P. We have shown that most of the dye was found in vagal afferent neurons containing substance P immunoreactivity. In the rats treated with perivagal capsaicin, there were no True blue-labeled cells in the nodose ganglia [24], which suggest that capsaicin treatment in this study was effective in ablating vagal sensory afferent function. Further, our previous electrophysiological studies have demonstrated that this treatment completely abolished the CCK [23,24], secretin-, and serotonin-elicited [18,36] vagal afferent neuronal response at physiological concentrations. Perivagal application of capsaicin also suppressed pancreatic secretion responses induced by CCK [20,24].

### Effects of CCK-A-receptor antagonist CR-1409

A polyethylene catheter (PE-10) was inserted into the external jugular vein as described above. It has been shown that in the anesthetized rat, the peptide antagonist CR-1409 abolished cerulein-stimulated pancreatic secretion in a dose-dependent manner. At a dose of 10 mg/kg, CR-1409 abolished pancreatic response to a near-maximum dose of cerulein [39], and blocked the vagal afferent responses to endogenous CCK stimulation [23]. In current studies, we examined the effects of the CCK-A-receptor antagonist CR-1409 (10 mg/kg intravenous bolus injection, dissolved in 0.005 N NaOH). CR-1409 was injected before administration of CCK-8 or luminal perfusion of peptone.

### Electrophysiological recording of ACC neurons

Detailed recording procedures have been described in our previous publications [4,26]. Briefly, anesthetized rat was place in a stereotaxic frame, an crania opening was made 1.0–5.0 mm anterior to bregma and 0.1–2.0 mm lateral to midline to record neurons in the ACC. Glass microelectrodes with tip diameters of 0.08 μm and 20–40 MΩ impedance were filled with neurobiotin and lowered into the rostral ACC by a micromanipulator (coordinates: 1.5–3.8 mm anterior to bregma, 0.3–1.0 mm lateral to midline, 1.5–3.5 mm ventral to brain surface). The rostral ACC, as defined by Vogt and Peters [5], is the area corresponding to perigenual Brodmann area 24b, portions of perigenual 24a, and caudodorsal area 32. After penetrating the surface of the cortex, the recording electrode was advanced until the spontaneous activity of a single unit could be accurately discriminated from the background neuronal noise. The signals were amplified by a high-input impedance preamplifier, displayed, and stored on a personal computer.

#### ACC neuronal activity in response to CRD

ACC neuronal spontaneous discharge was monitored for 2 min to confirm the stability of the basal firing frequency. The basal firing rate was assessed over 30 s to quantify the resting discharge in both control group and the rats infused with CCK-8. Every neuron isolated on the basis of spontaneous activity was studied to determine its response to CRD. The 50 mmHg CRD was produced by rapidly injecting saline into the balloon over 1 s and maintaining the distension for 30 s. A neuron was deemed responsive to CRD if its spike firing rate increased or decreased at least 10% from its pre-distension baseline activity. Neuronal discharge rates were measured 30 s before, 30 s during, and 120 s after CRD, with 5-min intervals in between, and evaluated on a time histogram (5-s bin width). In the control group, neurons responding to 50 mmHg CRD were tested twice to make sure the responses were consistent and repeatable [26]. In CCK-8 treatment group, after characterizing the CRD-excited ACC neurons, steady-state basal activity was recorded; then, each neuron was further tested in response to CRD with simultaneously infusion of saline and CCK-8, respectively.

#### Labeling and histological identification of recording sites: Juxtacellular injection

On completion of the experiment, recorded neurons were labeled by injecting them with neurobiotin by the technique of juxtacellular iontophoresis as we described previously [26].

### Visceromotor response (VMR) to colorectal distension (CRD)

To clarify whether administration of CCK-8 or intra-duodenal perfusion of peptone alters the visceral pain responses we measure visceral pain in animals based on brainstem reflexes, which have been described as “pseudoaffective” responses [25]. Details of this protocol were described in our previous publications [2,3,7]. Briefly, teflon-coated, 32-gauge stainless steel wires were implanted into the external oblique pelvic muscles to monitor the number of abdominal muscle contractions. 5 days after surgery graded-pressure CRD (0–20–40–60 mm Hg) was produced by rapidly injecting saline into the colonic balloon over 1 second and maintaining the distention for 20 seconds to establish stimulus response curves. The results of electromyography were quantified by calculating the area under the curve (AUC), which is the sum of all recorded data points multiplied by the sample interval (in seconds) after baseline subtraction.

### Statistical analyses

Statistical comparisons of the CPA data and VMR data among different treatment groups were made using two-way repeated-measures ANOVA followed by multiple comparisons adjusted by the Bonferroni test. For ACC neuronal firing, single neuronal responses were examined using Datapac 2000 (RUN Technologies, Mission Viejo, CA, USA). The prestimulus discharge frequency was assessed for 30s to quantify the resting discharge. The discharge frequency during CRD was also measured for 30s. The mean and standard deviation of ACC neuronal firing during the 30-s control period was compared with the activity after CRD. Data of spontaneous firing and firing rate to 50 mmHg CRD with saline and CCK-8 infusion were evaluated using Student’s t-test. Results were expressed as means ± SE. P < 0.05 was considered statistically significant.

## Abbreviations

ACC: Anterior cingulate cortex; AUC: Area under the curve; CCK: Cholecystokinin-octapeptide; CCK-RP: Cholecystokinin-octapeptide releasing peptide; CRD: Colorectal distention; CPA: Conditioned place avoidance; EA: Egg albumin; pACC: Perigenual; ACC: PrL, pre-limbic cortex; VH: Viscerally hypersensitive; VMR: Visceromotor response; VNS: Vagal nerve stimulation.

## Competing interests

The authors declare that they have no competing interests.

## Authors’ contributions

BC: electrophysiology study of ACC neuronal activity, technical support supervision; analysis and interpretation of data; XZ: animal surgery, VMR, interpretation of data and manuscript preparation; NY: conditioned place avoidance study, antagonist study; SC: perivagal application of capsaicin; YL: study concept, design, and supervision; analysis and interpretation of data, obtain funding and wrote the manuscript. All authors read and approved the final manuscript.
